# SIgA, TGF-β1, IL-10, and TNFα in Colostrum Are Associated with Infant Group B *Streptococcus* Colonization

**DOI:** 10.3389/fimmu.2017.01269

**Published:** 2017-10-20

**Authors:** Kirsty Le Doare, Katie Bellis, Amadou Faal, Jessica Birt, Daniel Munblit, Holly Humphries, Stephen Taylor, Fiona Warburton, Paul T. Heath, Beate Kampmann, Andrew Gorringe

**Affiliations:** ^1^Imperial College London, London, United Kingdom; ^2^Public Health England, Porton Down, United Kingdom; ^3^MRC Unit, Fajara, Gambia; ^4^Imperial College London, London, United Kingdom; ^5^I.M. Sechenov First Moscow State Medical University, Moscow, Russia; ^6^The In-FLAME Global Network, an Affiliate of the World Universities Network (WUN), West New York, United States; ^7^Public Health England, London, United Kingdom; ^8^St George’s University of London, London, United Kingdom

**Keywords:** breast milk, antibody, cytokines, neonatal immunity, microbiome, Group B *Streptococcus*

## Abstract

**Background:**

Group B *Streptococcus* (GBS) is a major cause of mortality and morbidity in infants and is associated with transmission from a colonized mother at birth and *via* infected breastmilk. Although maternal/infant colonization with GBS is common, the majority of infants exposed to GBS remain unaffected. The association between breastmilk immune factors and infant colonization and disease prevention has not been elucidated.

**Objectives:**

We have investigated the association between SIgA and cytokines in breastmilk and infant GBS colonization and clearance.

**Methods:**

Mother/infant GBS colonization was determined in a prospective cohort of 750 Gambian mother/infant pairs followed to day 89 of life. Anti-GBS secretory IgA bound to the surface of whole bacteria was assessed by flow cytometry and a panel of 12 cytokines quantified by mesoscale discovery in colostrum, breastmilk and serum.

**Results:**

Compared with infants receiving low anti-GBS SIgA in colostrum, infants receiving high anti-GBS SIgA were at decreased risk of GBS colonization for serotypes III and V. Infants colonized at day 6 were twice as likely to receive colostrum with high TGF-β1, TNFα, IL10, and IL-6 compared to uncolonized infants. Infants receiving high colostral TGF-β1, TNFα, and IL-6 had two-fold enhanced GBS clearance between birth and day 89.

**Conclusion:**

Our results suggest that the infant GBS colonization risk diminishes with increasing anti-GBS SIgA antibody in breastmilk and that key maternally derived cytokines might contribute to protection against infant colonization. These findings might be leveraged to develop interventions including maternal vaccination that may reduce infant GBS colonization.

## Introduction

Given the limited ability of newborns to respond efficiently to infectious agents, including at the mucosal surface, there is increasing interest in identifying maternal factors that may influence protection from infections in the early months of life. Neonatal immunity can be influenced by maternal factors through transfer of specific antibody across the placenta as well as *via* breastmilk (1). Several studies have shown that breastfed infants are better protected from infections of the gastrointestinal and respiratory tracts compared to formula-fed infants (2–4). The neonatal gut immune system is constantly modulated by colostrum and breastmilk, promoting the growth of certain bacteria over others and thereby influencing the neonatal immune response (1). Evidence now supports a connection between the mother’s gastrointestinal tract and the mammary glands *via* an “entero-mammary circulation,” which may play a role in immune priming (5).

One of the most important factors in breastmilk is secretory IgA (SIgA). SIgA mediates protection *via* binding to mucosal pathogens as well as neutralization of toxins and virulence factors. SIgA antibodies can prevent bacterial adhesion by binding to pili and other adhesins found on the surface of group B *Streptococcus* (GBS) (6, 7).

It is also established that cytokines in breastmilk vary according to maternal exposure to bacteria (8). TGF-β, IL-6, and IL-10 in breastmilk may enable the development and differentiation of neonatal cells that produce IgA (9) and are involved in the maturation of the neonatal gut immune system (10). It has also been shown that TGF-β1 and TGF-β2 in colostrum are positively correlated with infant IgA in neonatal serum (11, 12).

Little is known about the impact of cytokines and SIgA in breastmilk on bacterial colonization and clearance in general and in relation to GBS in particular. We tested the hypothesis that SIgA and breastmilk cytokines could influence infant colonization and clearance over the first three months of life in a cohort of Gambian infants.

## Materials and Methods

Samples were collected in an urban clinic in The Gambia, West Africa as part of a wider study of GBS infection previously reported (13). Ethical approval was given by the Gambia Government/MRC Joint Ethics committee (reference SCC 1350v4).

### Sample Collection

Sample collection and microbiology have been previously described (13). Briefly, 750 pregnant women between the ages of 18 and 40 years who had a low-risk, singleton pregnancy were recruited after giving informed consent. The study excluded very preterm (<32 weeks gestation) or very low birth weight (<2.0 kg) infants. Colostrum was collected within 12 h of birth and breastmilk was collected at day 6 and 60–89 days of life. After washing their hands, mothers wiped each breast with an alcohol wipe beforehand expressing 2–3 mL of colostrum and 4–5 mL of breastmilk. Milk samples were stored on cold packs at 4°C and transported to the laboratory within 6 h of collection. The lipid and whey layers were separated by centrifugation at 3,200 rpm for 30 min. The solid lipid layer was removed using a sterile scalpel and the whey stored separately in 1 mL aliquots at −70°C.

Midwives or field nurses collected rectovaginal swabs (Copan, UK) after cleaning of the perineum when women presented to the hospital in labor. Infant nasopharyngeal (calcium alginate swabs, Sterilin, UK) and rectal swabs (Copan, UK) were taken at 4 h of life, on day 6 and at day 60–89. Swabs were stored in 5 mL skim-milk tryptone glucose glycerol (STGG) on cold packs at 4°C and transported to the laboratory within 6 h of collection. The swab in STGG was vortexed briefly before being stored at −70°C (13).

### Microbiological and Molecular Quantification of GBS Colonization

The protocol was adapted from the Public Health England (PHE) protocol for the pre-incubation of swabs in enrichment broth before plating on solid media to enhance the yield of GBS (14). Swab samples were processed within 7 days of collection in batches of 100. Swabs in STGG were thawed on wet ice and transported to the microbiology laboratory. Samples were vortexed for 10–20 s and 100 µL of vortexed specimen was transferred to 2.5 mL Todd Hewitt Broth for 24 h pre-culture at 37°C in an atmosphere of 5% CO_2_. A Colombia Agar plate was divided into four segments, and 10 µL of neat suspension and each of three 1:10 dilutions were dispensed into each of the four quadrants and incubated overnight at 37°C in 5% CO_2_. From the primary plate, presumptive beta hemolytic Streptococcus colonies were streaked onto a blood agar plate and reincubated at 37°C in 5% CO_2_ for 24 h. Beta hemolytic streptococci appeared colorless or gray, about 2 mm in diameter with or without a surrounding zone of beta-hemolysis. Streptex^®^ (Oxoid, UK) was used in the qualitative identification of GBS. Samples were serotyped using PCR as previously described (13).

All infants were exclusively breastfed and all samples were collected before feeds to ensure samples were not contaminated with oral bacteria.

### Cytokine Quantification

The concentrations of cytokines IL-1β, IL-2, IL-4, IL-6, IL-10, IL-12, IL-13, IFN-γ, TNFα, and TGF-β1 were determined in the cord serum, colostrum, and breastmilk of a subset of 100 randomly selected Gambian mothers and serum from their infants at day 60–90, using electroluminescence *via* the Meso-Scale Discovery system and the Proinflammatory Panel 1 (human) MSD Multi-Spot Kit (MSD, Rockville, MD, USA). First, multi-analyte calibrator solution containing recombinant human cytokines at known concentrations, which have been expressed in *E. coli* or Sf21 insect cells, was reconstituted using the diluent provided. The calibrator was diluted fourfold six times to generate a series of seven reference calibrators to which unknown serum/breastmilk samples were compared. All serum/colostrum/breastmilk samples were diluted two-fold in the same diluent before addition to the plate. 50 µl of the diluted serum or breastmilk and calibrators were added to each well. Plates were incubated at room temperature for 2 h and then washed three times with phosphate-buffered saline (PBS) containing 0.05% Tween-20. 25 µl of a solution containing detection antibody to each cytokine was added to each well. Plates were then incubated for a further 2 h at room temperature. After the incubation, plates were washed and 150 µl read buffer added. Plates were then read using the MESO QuickPlex SQ 120 MSD instrument. Samples below the limit of quantification were allocated a value for each cytokine of half the lower limit of detection as per manufacturer’s instructions. Details of each lower limit of quantification and the associated allocations for analysis are found in Table S2 in Supplementary Material. The coefficient of variance (CoV) for the assay was 25%.

### SIgA Binding to GBS Bacteria

The assay was adapted from a serological assay described by Le Doare et al. (15) which quantified IgG binding to GBS bacteria using serum samples. GBS isolates used in this study were H092040676 (ST Ia), H092120162 (ST III), and H091780506 (ST V), which were kindly provided by Professor Androulla Efstratiou, PHE, Colindale. 5 µl of the breastmilk sample was added to a 96 well micro-titer plate and incubated with 195 µl of killed GBS of STs Ia, III, and V diluted to OD 0.05 (at 600 nm) in PBS with 2% bovine serum albumin (BSA) for 30 min at 25°C while shaking at 900 rpm. After centrifuging at 3,060 *g* for 5 min, excess fluid was removed. The pellet was washed with 200 µl of PBS with 2% BSA before centrifuging. The pellets were then re-suspended in rabbit anti-human SIgA polyclonal FITC antibody (BIOSS, Woburn USA) diluted to 1:100 in PBS with 2% BSA. This was incubated at 4°C for 20 min. After centrifugation, the pellet was washed twice. The final pellet was re-suspended in PBS with 1% formaldehyde to kill any sample bacteria in the breastmilk before SIgA binding was analyzed using a Beckman Coulter Cyan flow cytometer with attached Cytek 96 well plate loader (High Wycombe, UK) calibrated with Ultra rainbow beads (San Diego, CA, USA). A secondary gate was set to 10% of the histogram of the bacteria and conjugate wells providing a percentage gated value for each of the samples. The percentage gated and the mean fluorescence for that population were multiplied together to give a fluorescence index (FI). The FI of the conjugate control was subtracted from each sample’s mean Fl to remove background, non-specific binding. This is referred to as florescence intensity (FI-C) (see Figure S1 in Supplementary Material). For the measurement of SIgA deposition, raw data were plotted and used in the analysis. Where no antibody deposition was detected, these data were given a value of ND and excluded from analysis; all samples were run in duplicate and samples were accepted if the standard error was < 5%. Over the course of the study, the mean SD of FI for zymozan controls was 8,831 (CoV, 31.6%).

### Statistical Analysis and Sample Size Calculation

To ensure that the study avoided bias, we followed the statistical design of experiments for cluster analysis method (16). Based on estimates from the original study in The Gambia [21% infants colonized (158/750); 6.5% prevalence of the lowest serotype GBS STIa] (13) a sample size of 750 women would be required to give at least ten women/5 infants colonized with GBS STIa. Five infants for each GBS ST would give 80% power to detect a correlation of 70% or greater between GBS ST-specific antibody and colonization.

STATA version 12 (StataCorp 2013, California, CA, USA) and GraphPad Prism version 6.0 (GraphPad Software Inc., La Jolla, CA, USA) were used for statistical analysis. Additional statistical support was provided by Fiona Warburton (PHE) who verified all statistical analysis.

Potential differences between cytokines in colostrum and breastmilk were calculated using the Mann–Whitney *U* Tests or Wilcoxon Rank sum tests. Radar plots were generated to demonstrate the distribution of cytokines between colonization groups. Odds ratios, adjusted odds ratios, and analysis of variance (ANOVA) were calculated to compare groups. Potential differences in antibody concentrations between colonized and non-colonized mothers and infants were evaluated by Student’s *t*-test after log transformation of data. Three groups were compared (mother colonized/infant non-colonized; mother colonized/infant colonized; and neither mother nor infant colonized) using ANOVA following log-transformation of data. Comparison of log-transformed serum FI-C values at day 6 and 60–89 days was performed using a paired *t*-test. Linear regression was calculated from log transformed data to determine IgG in serum and IgA in breastmilk.

### Definitions

Maternal colonization was defined as the identification of a GBS-positive rectovaginal swab at time of delivery. Infant colonization was defined as the identification of a GBS-positive nasopharyngeal and/or rectal swabs at 4 h (birth), day 6 (early colonization), or day 60–89. We identified infants colonized both at birth and day 6 as the baseline colonization point to ensure we captured only true colonization episodes. Infant acquisition of colonization was defined as a positive nasopharyngeal and/or rectal swab at day 6 and/or day 60–89 when the associated swab from the previous visit was negative, and loss was defined as a negative nasopharyngeal and/or rectal swab when the previous swab was GBS-positive. Persistently colonized infants were defined as infants where nasopharyngeal and rectal swabs at birth, day 6, and day 60–89 were all GBS positive.

## Results

The maternal GBS colonization rate was 32% (*n* = 237), with 21% of infants colonized at birth (*n* = 158), 20% colonized at day 6 (*n* = 152), and 7% of infants colonized on day 60–89 (*n* = 50) (13). 680 colostrum and 750 breastmilk samples were available for analysis.

We examined the presence of GBS in breastmilk from all 750 women and found 10 to be GBS colonized in breastmilk.

### Relationship between SIgA in Breastmilk and Maternal GBS-Colonization Status

Overall, 324/680 (47.6%) colostrum samples had detectable SIgA against any GBS ST. There was no significant difference in SIgA concentration between non-colonized mothers and those colonized with STs Ia [GBS + mother FI-C 1300 (1,000–1765) GBS-mother 1500 (1000–1886) *p* = 0.3], III [GBS + 1713 (1,545–4,837), GBS-1995 (1,798–2,223) (*p* = 0.3)], or V [GBS + 2089 (1,883–5,896) GBS-2691 (2,426–2,985) (*p* = 0.8)].

There was no difference in IgA antibody concentrations found between those women colonized in breastmilk and those uncolonized.

### Relationship between SIgA in Colostrum and Maternal/Infant Colonization on Day 6

**Figure 1:**
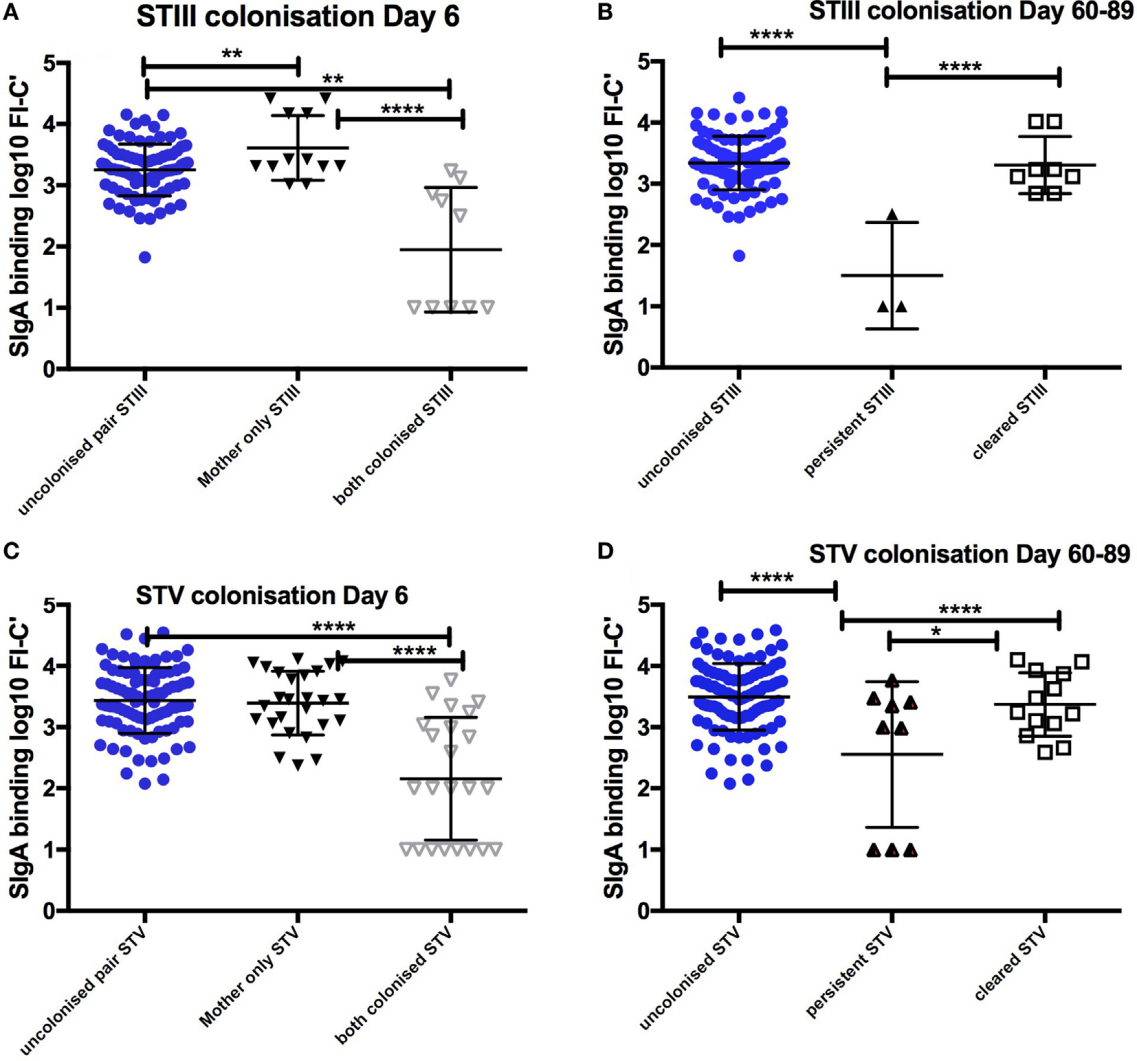
Relationship between SIgA in colostrum and maternal and infant colonization group for STIII and STV. Mean and SD of log transformed florescence intensity (FI-C) of SIgA deposition onto the surface of whole group B *Streptococcus* (GBS) bacteria STIII and STV at day 6 **(A,C)** and clearance of colonization between day 6 and day 60–89 **(B,D)**. STIII (*n* = 3 persistent, *n* = 12 cleared colonization, *n* = 106 non-colonized any serotype) STV (*n* = 9 persistent colonization, *n* = 33 cleared colonization, *n* = 106 non-colonized any serotype). *p < 0.05, **p < 0.01, ****p < 0.0001. Table S1 in Supplementary Material outlines the numbers in each group by colonizing serotype.

152 infants [152/680 (22.4%)] were colonized both at birth and on day 6 of life. Colonized infants born to colonized mothers received colostrum with lower SIgA binding to GBS than any other colonization group. Non-colonized infants born to colonized mothers received colostrum with significantly higher SIgA than colonized mother/infant pairs for STIII and STV, when adjusted for maternal age, maternal weight, maternal anemia, and gestation (*p* < 0.0001) (Figure 1).

### SIgA in Colostrum Is Associated with Absence of Infant Colonization at Days 60–89

Compared to infants who remained colonized on day 60–89, non-colonized infants received higher SIgA concentrations against STV in colostrum (*p* < 0.001). There were no infants who remained colonized with STIa and only three infants remained colonized with STIII. Infants who cleared colonization between birth and day 6 also received colostrum with higher concentrations of SIgA than those infants who remained colonized for STV (Figure 1). In addition, non-colonized infants at 60–89 days were more likely to have higher antibody concentrations in cord blood and in infant serum (*p* < 0.001). There was a positive correlation between SIgA in colostrum and complement-mediated antibody deposition in cord blood and in infant serum found in the main study (*R* = 0.67, *p* < 0.0001) (17).

### Cytokines in Colostrum, Breastmilk, and Infant Serum

**Figure 2:**
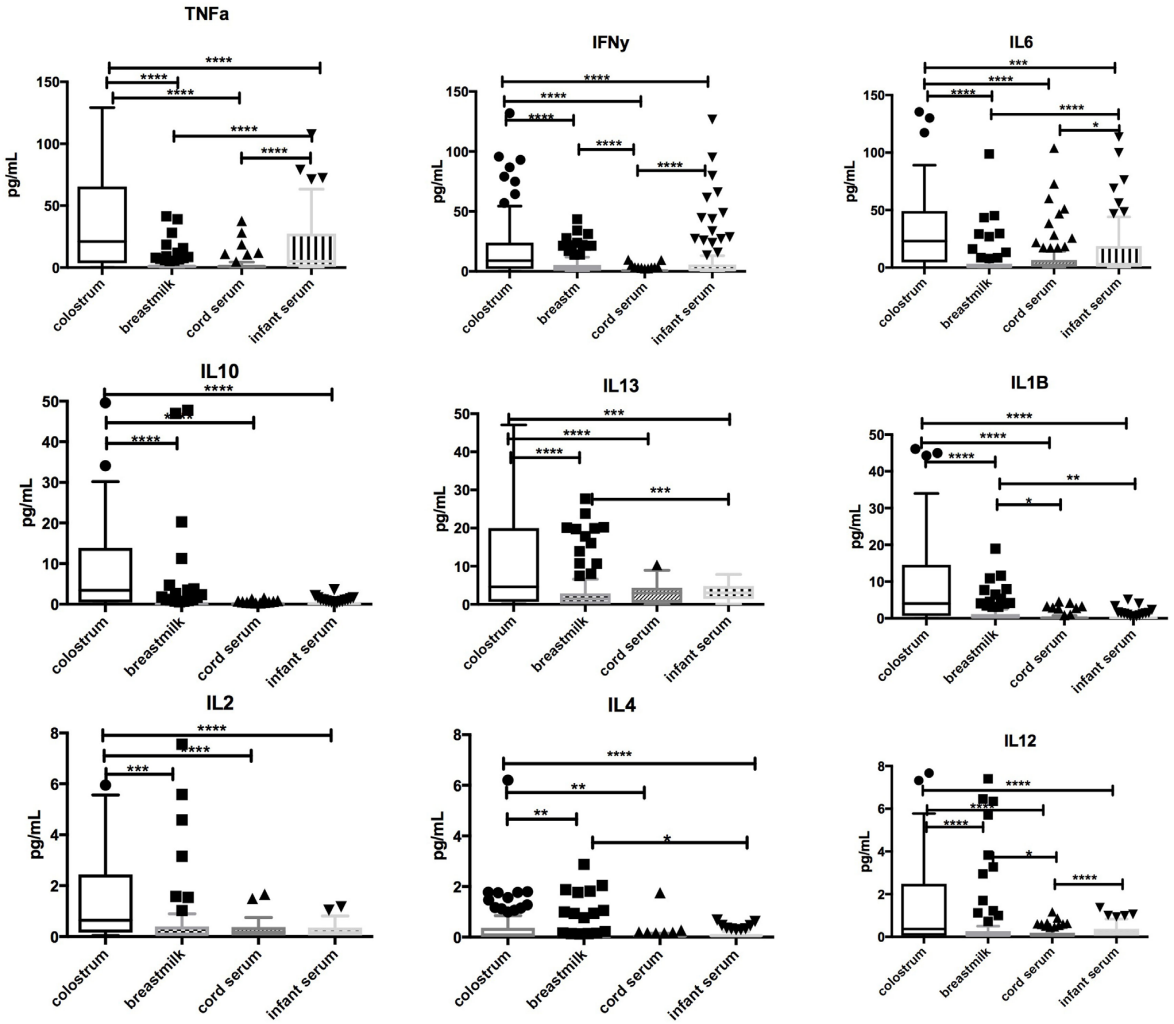
Comparison of cytokine concentrations in colostrum, breastmilk, cord blood, and infant serum. Box-and-whisker plot of concentrations of TNFα, IFN-γ, IL-1β, IL-2, IL-4, IL-6, IL-10, IL-12, and IL-13 in colostrum, breastmilk, cord, and infant serum in picograms per milliliter (pg/mL). 100 samples were assessed. Median and interquartile range is demonstrated. Solid shapes indicate outliers. *p = <0.05, **p < 0.01, ***p < 0.001, ****p < 0.0001.

Significantly higher concentrations of TNFα, IFNγ, IL-1β, IL-2, IL-4, IL-6, IL-10, IL-12, IL-13 (*p* < 0.0001 for all) were found in colostrum in comparison with breastmilk, cord blood, or infant serum at days 60–89. Breastmilk had lower concentrations of TNFα, IL-1β, IL-6, and IL-13 (*p* < 0.0001) than infant serum. Low levels of IL-2, IL-4, and IL-12 were found in all samples. Associations between cytokines in colostrum, breastmilk, cord blood, and infant serum are highlighted in Figure 2.

### Relationship between Cytokines in Colostrum and Early Infant Colonization

**Figure 3:**
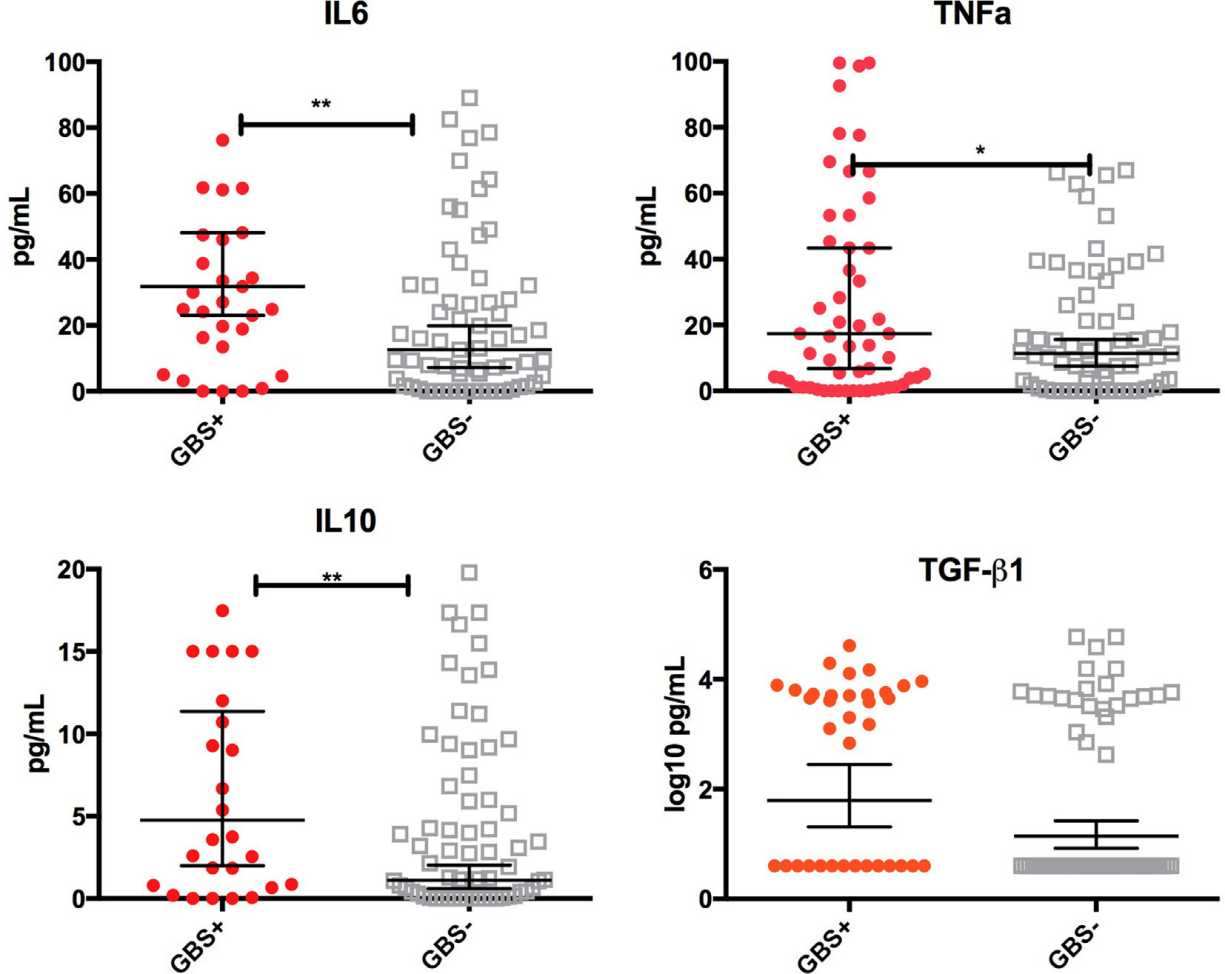
Cytokine concentrations in colostrum by infant group B *Streptococcus* (GBS) colonization status at day 6. Box-and-whisker plot of colostrum concentrations of TNFα, IL-6, IL-10, and log_10_ values of TGF-β1 in picograms per mililitre (pg/mL). 65 of the 100 samples had cytokine concentrations above the lower limit of detection. Geometric mean and 95% confidence interval is demonstrated. Solid shapes indicate outliers. *p = <0.05, **p < 0.01.

Infants who were still colonized on day 6 of life were more likely to receive colostrum with high concentrations of TGF-β1 [OR 1.45 (1.1–1.9), *p* = 0.02], IL-10 [2.8 (1.1–7.5), *p* = 0.05], TNFα [2.4 (1.1–5.0), *p* = 0.02], and IL-6 [2.4 (1.2–5.0), *p* = 0.02] than non-colonized infants (Figure 3). Multivariate logistical regression adjusted for other cytokines showed that colonized infants were more likely to receive colostrum with high concentrations of TGF-β, IL-10, TNFα, and IL-6 than non-colonized infants [AOR 3.2 (1.8–9.2), *p* = 0.03]. There was no association with any other cytokine at day 6.

### Relationship between Cytokines in Colostrum and Acquisition and Clearance of Infant Colonization between Birth and Days 60–89

**Figure 4:**
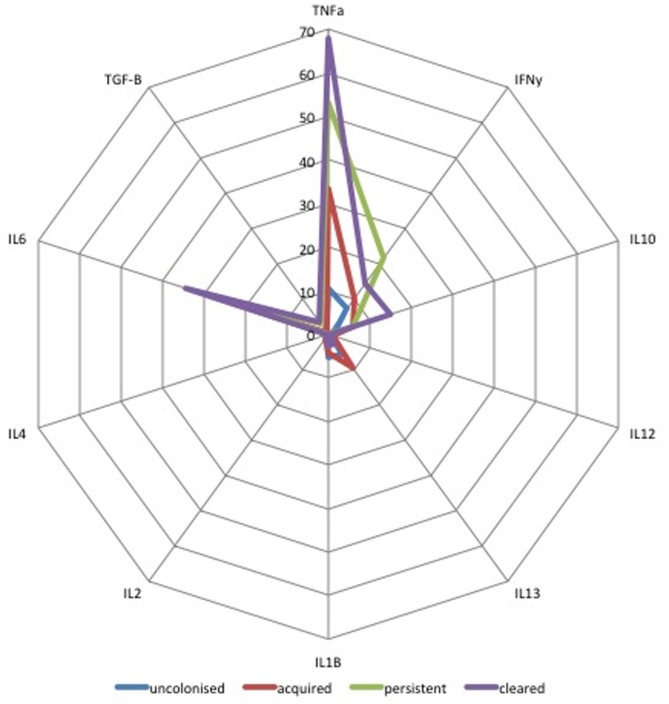
Radar plot of cytokine concentration in colostrum by group B *Streptococcus* (GBS) colonization status by colonization status of infants. Radar plot of median cytokine concentration in colostrum in pg/mL from: blue = non-colonized, green = persistently colonized infants, red = infants who acquired colonization and purple = infants who cleared colonization between birth and day 60–89. TGF-β values are shown as log_10_ pg/mL values.

Median concentrations of ten cytokines in colostrum were compared for all four mother/infant groups. As shown in Figure 4, infants who cleared colonization between birth and days 60–89 (*n* = 27) received colostrum with higher concentrations of TNFα, IL-6, and TGF-β1 (*p* = 0.01) than infants who acquired colonization between birth and day 60–89 [AOR 2.4 (1.2–5.0), *p* = 0.02]. Infants who were persistently colonized (*n* = 7) received higher concentrations of IFN-γ in colostrum than infants who acquired colonization (*n* = 28) [AOR 5.1 (2.4–11.0), *p* < 0.001]. There was no association between other cytokines in breastmilk and infant colonization at day 60–89 (Figure 4).

## Discussion

The results from our large cohort of mother/infant pairs show for the first time that SIgA and key immunomodulatory cytokines TNFα, IL-6, IL-10, and TGF-β in colostrum may be associated with infant GBS colonization, acquisition, and clearance up to 3 months of life.

Few studies have focused on the relationship between SIgA in breast milk and infant colonization with potential pathogens. Given that infant GBS colonization is the pre-requisite for disease, investigating factors that can reduce infant colonization is important, as manipulation of such factors could reduce the risk of invasive disease. Our data, obtained using an assay that measures SIgA that binds to whole GBS bacteria of STs Ia, III, and V, demonstrate that compared to all other mother/infant groups, the lowest anti-GBS SIgA levels were found in colonized mother/infant pairs. We also show that infants receiving the lowest concentration of anti-GBS SIgA in breastmilk were less likely to clear GBS colonization. Several studies have investigated anti-GBS antibody levels in breastmilk. Lagergard et al. (1992) identified IgA antibodies to CPS type III GBS in 63% of a cohort of 70 Swedish women (18), while Weisman and Dobson (1991) determined anti-STIa, II or III CPS IgG in a cohort of 46 USA women and found levels at approximately 10% of those in maternal serum (19). The most recent study of SIgA was conducted by Edwards et al. (2004). The study investigated IgG and IgA in breast milk to GBS ST III CPS in 9 colonized and 9 non-colonized women with antibody titers less than or equal to 1 µg/mL and those greater than 1 µg/mL and also found that detectable levels of anti-STIII SIgA in breastmilk correlated with high levels in maternal serum (20).

In line with our own observations, animal models of GBS ST Ib and III disease (21–23) demonstrate an association between increased SIgA in breastmilk and pup survival. There have been few studies of the effect of breastmilk antibody on infant colonization, but a small study of breastfed infants challenged postnatally with non-pathogenic *E. coli* demonstrated a reduction in colonization with high breastmilk SIgA concentrations (24), whilst studies of *H. influenzae* (25), *S. pneumoniae* (26), and *N. meningitides* (27) demonstrate that increasing antibody concentrations in serum are associated with reduced risk of pharyngeal colonization with these bacteria. If the same is true of SIgA in breastmilk and GBS colonization, then increasing maternally derived antibody in breastmilk in conjunction with increasing serum antibody through vaccination could interrupt GBS colonization and subsequently development of GBS disease.

The highest concentration of SIgA antibody in milk was found in mother/infant pairs where the mother but not the infant was colonized. This could be explained by the development of an immune response in the mother, triggered by a new GBS challenge. This antibody is then passed to the infant and acts in a protective fashion. Timing of acquisition of maternal GBS colonization is likely to be important but our study was not able to assess this aspect. The fact that mother/infant pairs who remain completely non-colonized also have high concentrations of anti-GBS SIgA antibody indicates a long half-life of IgA during lactation due to previous colonization, as has been seen in mothers vaccinated with *N. meningitidis* vaccines (28). Alternatively, this could also be due to another cross-reactive antigen that we have not identified.

Our finding of the association between TNFα and *de novo* GBS colonization fits with the results of the only other published study of breastmilk cytokines and infant disease by Riskin et al. (2012). This study analyzed the breastmilk of exclusively breastfeeding mothers of 31 sick infants under 3 months of age and demonstrated that increases in TNFα in colostrum were associated with acute infant disease, whether or not the mother was also sick (29). A possible explanation is that colostral TNFα induced an increase of MHC class II expression on antigen presenting cells (30) and expression of receptors that facilitate transcytosis of SIgA into exocrine fluids in the respiratory and gastrointestinal tracts to counteract infection (31). *In vitro* studies have similarly demonstrated that increased TNFα was associated with altered binding of *E. coli* in intestinal cells (32). If the same is true with GBS, then increased colostral TNFα could reduce GBS binding to neonatal gut epithelium and reduce the risk of systemic invasion by inducing IgA at the mucosal surface.

Our observation that early infant colonization was associated with increased concentrations of IL-10 and TGF-β might be explained by IL-6-driven synthesis of IgM and IgG together with TGF-β as part of the maternal immune response to a pathogen during active infection, which equally increases IgA synthesis from naive B cells (33). However, it is also possible that the cytokines in breastmilk result from local immune activation in the mammary glands due to GBS in breastmilk. These hypotheses remain speculative at present and future longitudinal studies of breastmilk and assessment of function of infant gut epithelial cells in response to pathogen challenge would be required to provide more insight into this phenomenon.

Our study has several limitations. As cytokines vary within and between mothers, our data can only provide a cross-sectional snapshot and a larger cohort with longitudinal maternal sampling is needed to fully appreciate the implications of these results. On average 10 of the 100 samples analyzed for each cytokine had levels below the limit of detection of our assay and we have allocated an arbitrary level of half the lower limit of detection into our results as the most robust way of dealing with these data. There is the possibility that this may introduce bias into our results but this is reduced by the analysis of logged data.

Additionally, the data surrounding the associations between acquisition and loss of infant GBS colonization do not take into account maternal colonization at each time point, as this was only measured in mothers at delivery. Maternal colonization is transient (34) and we cannot be certain that mothers who were not colonized at birth did not acquire GBS colonization themselves during the study. The collection of repeat rectovaginal swabs from the mothers in addition to breastmilk and infant samples was beyond the scope of our study. In future studies, it might be possible to consent for longitudinal swabbing in order to better understand the dynamics between maternal colonization, breastmilk SIgA, cytokine production and subsequent infant colonization over time.

We did not conduct any analysis of the association between cytokines and SIgA antibody and GBS colonization as the complexity of the model and the sample size needed was outside of the scope of this study. However, we have demonstrated that infants who remained non-colonized received high concentrations of SIgA in colostrum and high functional antibody in cord blood and infant serum (17), suggesting a role for both systemic and mucosal antibody in preventing GBS colonization.

The low numbers of mother/infant pairs colonized with GBS STIa and III precluded detailed analysis of these GBS serotypes, which are important causes of infant disease in many countries and represent two of the serotypes being targeted by the current vaccine candidates. The serotype distribution in The Gambia is different from that in the USA and Europe, and highlights the need to understand GBS serotype prevalence in different regions (13).

The lack of standardized protocols available for the measurement of specific SIgA in breastmilk remains a limitation not just of this study, but in general, restricting comparison with other studies. Going forward, it will be very important for the field that assay standardization is undertaken in order to demonstrate consistency in our predictions of antibody-mediated protection from colonization and disease between studies, particularly when assessing novel vaccines against GBS and their potential impact on breastmilk factors.

We did not seek to measure cellular or functional mucosal immunity as part of our cytokine analysis, therefore the data do not offer mechanistic insights into the role of cytokines in breastmilk and interactions at the mucosal surface.

Finally, we made the assumption that the reduction in colonization is due to breastmilk cytokine and SIgA antibody concentrations. It is probable that protection from colonization at the mucosal surface is more complicated and includes both innate and adaptive factors associated with breastmilk (35) or the infants’ developing microbiome (1). Measurement of the complexity of breastmilk immunity is extremely challenging, as there are few methods available and to assess these additional aspects requires sophisticated phenotyping of breast milk immune cells. However, samples have been stored for a comprehensive proteomic interrogation of breastmilk components and further work is ongoing.

In conclusion, our data support the notion that the risk of GBS colonization in infants diminishes as naturally acquired SIgA antibody in breastmilk increases as part of the maternal immune response to GBS, and that the presence of key cytokines such as TNFα, IL10, and TGF-β might further contribute to protection. Our findings support the idea that increasing SIgA through vaccinating mothers in pregnancy against GBS could have the same effect, provided the antibody levels and function induced by vaccination are similar or greater than that of naturally acquired antibody.

## Ethics Statement

This study was carried out in accordance with the recommendations of the MRC Gambia joint research ethics committee with written informed consent from all subjects. All subjects gave written informed consent in accordance with the Declaration of Helsinki. The protocol was approved by the MRC Gambia joint research ethics committee SCC1350v4.

## Author Contributions

KLD was responsible for original data, study design, statistical analysis, and manuscript writing; KB, AF, and JB were responsible for the laboratory analysis and had input into the manuscript writing; DM, HH, and ST were responsible for overseeing the laboratory analysis and had input into study design and the final manuscript; FW was responsible for statistical analysis plan and the overall statistical analysis. PTH, AG, and BK had intellectual input into study design and final manuscript. All authors had access to the data.

## Conflict of Interest Statement

KLD, KB, AF, JB, FW, HH, ST, and AG declare no conflict of interests. PTH is an advisor to Pfizer and GSK vaccines; BK is an advisor to Pfizer and GSK vaccines. The MRC Unit The Gambia has previously received funding for vaccine trials, including vaccines produced by Pfizer and GSK.

## Abbreviations

GBS, group B *Streptococcus*; SIgA, secretory Ig A; FI-C, fluorescence intensity; STGG, skim-milk tryptone glucose glycerol; ST, serotype; PBS, phosphate buffered saline; BSA, bovine serum albumin.
