# Supplementary material for: SIgA, TGF-β1, IL-10, and TNFα in Colostrum Are Associated with Infant Group B Streptococcus Colonization
Source: Front Immunol. 2017 Oct 20;8:1269. doi: 10.3389/fimmu.2017.01269 (PMC5660603; doi:10.3389/fimmu.2017.01269)
Supplement: Supplementary file 1 [file Data_Sheet_1.docx]

**Supplementary table 1- number of samples with detectable IgA deposition against any serotype.**

|  | STIa | STIII | STV |
| --- | --- | --- | --- |
| Mother only | 5 | 12 | 27 |
| Mother and infant day 6 | 2 | 11 | 23 |
| Infant persistently colonised | NA | 3 | 9 |
| Infant cleared colonisation | NA | 8 | 14 |

**Supplementary Table 2 – lower limit of detection of cytokines in MSD assay and value assignment for analysis**

|  | Lowest Limit of Detection (pg/ml) | Value assigned |
| --- | --- | --- |
| IL-1β | 0.04 | 0.02 |
| IL-2 | 0.09 | 0.045 |
| IL-4 | 0.02 | 0.01 |
| IL-6 | 0.06 | 0.03 |
| IL-10 | 0.03 | 0.015 |
| IL-12p70 | 0.11 | 0.055 |
| IL-13 | 0.24 | 0.12 |
| TNFα | 0.04 | 0.02 |
| IFNγ | 0.2 | 0.1 |
| TGF-B1 | 8.73 | 4.365 |
| TGF-B2 | 265 | 132.5 |
