# Supplementary material for: SIgA, TGF-β1, IL-10, and TNFα in Colostrum Are Associated with Infant Group B Streptococcus Colonization
Source: Front Immunol. 2017 Oct 20;8:1269. doi: 10.3389/fimmu.2017.01269 (PMC5660603; doi:10.3389/fimmu.2017.01269)
Supplement: Supplementary file 2 [file Data_Sheet_2.pdf]

Supplementary figure 1 – example of gating of GBS bacterial samples using flow cytometry

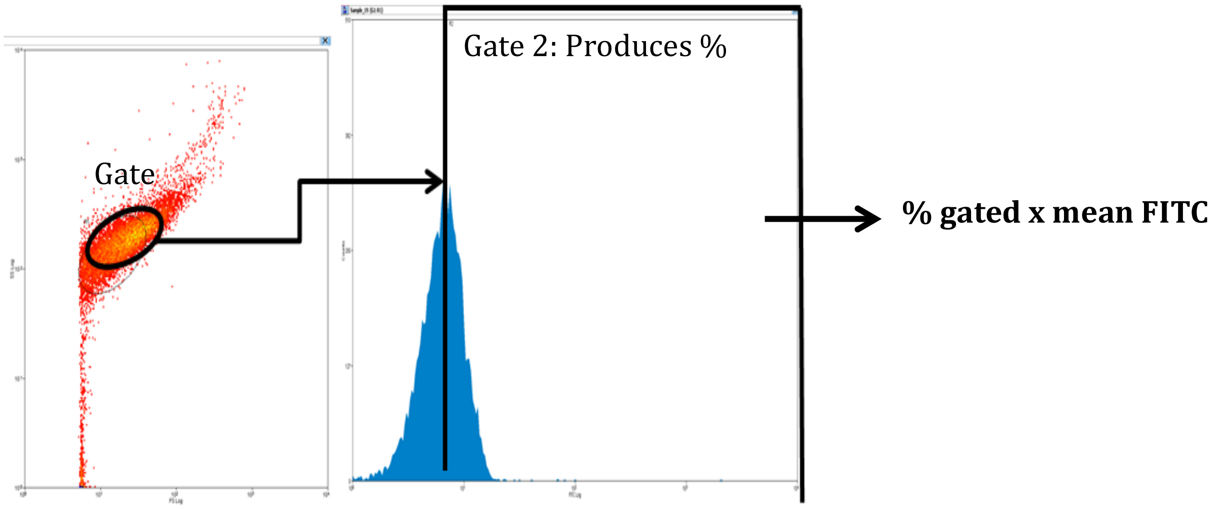

Diagram outlines gating of bacterial population, followed by gating to include 10% of the bacteria plus conjugate control followed by calculation of the fluorescence index and then subtracting the conjugate only control to give a final value FI-C' for the test sample.
